# Fumonisin B1 exposure induces cardiac inflammation in C57BL/6 mice

**DOI:** 10.1007/s00204-026-04342-x

**Published:** 2026-03-31

**Authors:** Selwyn Gounder, Ndumiso Mhlongo, Terisha Ghazi, Anil Chuturgoon

**Affiliations:** Discipline of Medical Biochemistry, School of Medicine, University of KwaZulu-Natal, Durban, South Africa

**Keywords:** Fumonisin B1, Mice heart, Inflammation, DNA methylation, TNF-α, NF-κB

## Abstract

The increasing prevalence of mycotoxin toxicity poses significant health risks, contributing to various diseases. Among these, fumonisin B1 (FB_1_) alters sphingolipid biosynthesis, induces oxidative stress, apoptosis, mitochondrial dysfunction, and inflammation. This study investigated the impact of acute FB_1_ exposure on inflammation and epigenetics changes in hearts of C57BL/6 mice. Molecular docking was performed to identify potential interactions between FB_1_ and key inflammatory proteins (TNF-α, iNOS, NF-κB p65, and NF-κB p50). Excised C57BL/6 mice heart tissue was analysed for gene expression (qPCR), protein expression (Western blotting), nitric oxide levels (NOS assay), cytokine levels (ELISA), and global DNA methylation (ELISA). Molecular docking suggested FB_1_ interacted with key residues in TNF-α, iNOS, and NF-κB, potentially influencing their activity. Gene expression analysis (*TNF-α, NF-κB, IL-6, NLRP3 Inflammasome, IL-18, caspase 1, IL-1β, GSDMD, caspase 3, CT-1, IL-10, MBD2, DNMT1, DNMT3A, and DNMT3B*) revealed that FB_1_ significantly dysregulated inflammatory cytokines and DNA methylation-related genes. Protein expression analysis showed significant upregulation of pro-inflammatory cytokines (TNF-α, NF-κB, IL-6, IL-1β, IL-18, IL-10, and TGF-β1). Global DNA methylation levels were significantly increased, with notable upregulation of DNMT1. In conclusion, acute exposure of C57BL/6 mice to FB_1_ significantly impacted inflammatory and DNA methylation pathways, leading to cardiac distress complications.

## Introduction

The increasing prevalence of mycotoxin toxicity poses a significant healthcare burden, contributing to a variety of diseases. Over 500 mycotoxins have been identified in recent years (Awuchi et al. 2022). Among these, the sphingosine/sphinganine analogue mycotoxin, fumonisin B1 (FB_1_), has garnered substantial research interest. FB_1_, a member of the fumonisin family, is a water-soluble mycotoxin produced by *Fusarium verticilliodes* and *Fusarium proliferatum* (Anumudu et al. 2024). Health-related risks associated with FB_1_ toxicity have been documented in both animals and humans. Extensive research has demonstrated FB_1_'s ability to induce diseases in animal models, including hepatitis in broilers, turkeys, and ducks; leukoencephalomalacia in horses; porcine pulmonary oedema; and potent liver and kidney carcinogenesis in rats and mice (Marasas et al. 1988; Haschek et al. 2001; Gelderblom et al. 1994).While these studies have provided preliminary associations between FB_1_ and human health risks, FB_1_ has been shown to induce complex pathways particularly inflammation (Gounder et al. 2025).

Inflammation is a vital process that mitigates the harmful effects of toxins, mechanical injuries, and microorganisms. It involves the recruitment of white blood cells such as neutrophils, macrophages, basophils, eosinophils, and lymphocytes (Shi and Pamer 2011). The inflammatory pathway is activated when foreign entities such as toxins like FB_1_, foreign cells, and tissue damage trigger a cascade of mediators that induce an inflammatory response. The genes responsible for coordinating the inflammatory response encode mediators, known as pro-inflammatory cytokines and chemokines, such as (Tumour necrosis factor alpha) TNF-α, (inteleukin-1-beta) IL-1β, (interleukin 18) IL-18, and (interleukin 6) IL-6 (Zhang and Dhalla 2024). Among these, TNF-α and IL-1β are potent inflammatory molecules, primarily functioning in acute inflammation. Stimulation of TNF-α by toxins can activate the (Nuclear Factor kappa-light-chain-enhancer of activated B cells) NF-κB pathway, which regulates the expression of inflammatory genes for cytokines and molecules such as IL-1β, IL-6, IL-8, and inducible nitric oxide synthase (iNOS) (Tripathi and Aggarwal 2006). The IL-6 cytokine family includes cardiotrophin-1 (CT-1), which can mitigate a pro-inflammatory environment by inhibiting TNF-α and degrading IκB, an inhibitory factor of NF-κB, thus playing a role in NF-κB pathway activation (Jougasaki 2010). The enzyme iNOS, activated after NF-κB transcription, produces nitric oxide (NO) from the L-arginine pathway. At low concentrations, NO inhibits cytokines and chemokines, but at higher concentrations, it can aggravate the pro-inflammatory response and cause tissue damage (Laroux et al. 2001; Guzik et al. 2003). Increased NF-κB activity also triggers the transcriptional activation of inflammasomes, specifically the (nucleotide-binding oligomerization domain (NOD) like receptor family pyrin domain containing 3) NLRP3 inflammasome. Activation of the NLRP3 inflammasome, influenced by the pro-IL-1β signal, leads to caspase 1 activation, which cleaves pro-IL-1β and pro-IL-18 into their active forms, further amplifying the inflammatory response (Franchi et al. 2009). Caspase 1 activation can also trigger pyroptotic cell death by cleaving gasdermin D (GSDMD) into its N-terminal form (N-GSDMD), creating pores in the cell membrane. Similarly, increased caspase 3 activity can cleave gasdermin E (GSDME) at the N-terminal, leading to pore formation and pyroptotic cell death (Zmora et al. 2017; Tsuchiya et al. 2019; Jiang et al. 2020). These mediators exacerbate inflammation by altering vascular permeability, leading to neutrophil recruitment to the injury site. The duration of inflammation depends on the injury’s severity and is often associated with excessive pro-inflammatory cytokine activation (Ahmed 2011). To mitigate the harmful effects of an altered inflammatory response, the anti-inflammatory cytokine (interleukin 10) IL-10 is stimulated to limit inflammation. IL-10 inhibits macrophages at the injury site, decreasing pro-inflammatory cytokines, and suppresses antigen presentation in dendritic cells, attenuating the pro-inflammatory response (Steen et al. 2020). Another anti-inflammatory cytokine, (Transforming growth factor beta 1) TGF-β1, regulates apoptosis, cell growth, and differentiation. When pro-inflammatory cytokines such as TNF-α activate the NF-κB pathway, they can induce TGF-β1 expression (R Skeen et al. 2012). The expression of TGF-β1 suppresses pro-inflammatory TNF-α and IL-1β, creating a more anti-inflammatory environment (Kim et al. 2021). As mentioned above these mediators are both transcriptionally and post-transcriptionally thus, key processes such as DNA methylation could affect the activity of these mediators.

Epigenetics is an evolving field characterised by heritable changes in gene expression that do not involve mutations or alterations in the gene sequences. Instead, these changes rely on modifications to the external structure of DNA. One of the most studied epigenetic mechanisms is DNA methylation, whereby a methyl group is added to the 5’ position of cytosine residues, forming 5-methylcytosine (5mc) (Singal and Ginder 1999). This process is governed by enzymes called DNA methyltransferases (DNMTs), including DNA Methyltransferase 1 (DNMT1), DNA Methyltransferase 3 Alpha (DNMT3A), and DNA Methyltransferase 3 Beta (DNMT3B). DNMT1 maintains DNA methylation during replication, ensuring the methylation pattern from the parent strand is copied to the new strand. In contrast, DNMT3A and DNMT3B establish new methylation patterns on unmethylated strands. Adding these methyl groups prevents proteins from recognising specific gene sequences, thereby repressing gene expression (Hamilton 2011; Gibney and Nolan 2010; Bird 2002). Many animal studies have shown FB1’s harmful effects on various tissues and organs, particularly the kidneys, liver, and gastrointestinal tract. However, there is limited data on its effects on heart tissue. Among the few studies on FB1’s cardiotoxicity, it has been shown to block sphingosine-mediated L-type calcium channels, leading to heart failure (Constable et al. 2000). Additionally, FB1 can decrease myocardial contractility and arterial blood pressure, however, the specific mechanisms of action remain unclear. Therefore, understanding the mechanism of FB1 in cardiac tissue is crucial. Principally, inflammation and epigenetics are potential pathways linking FB1 to cardiac distress. Therefore, the aim of this study was to determine if acute 24-h FB_1_ exposure on C57BL/6 mice leads to alterations in inflammation and DNA methylation prompting cardiac distress.

## Materials and methods

### Molecular docking

The 2D structure of FB_1_ was obtained from the PubChem (CID: 2,733,487). The geometry of the FB_1_ structure was energy minimized using the General Amber Force Field (GAFF) with Steepest Descent algorithm implemented in Avogadro version 1.2.0 (Hanwell et al. 2012). The structure was then saved in a 3D Mol2 format. The structures of the proinflammatory factors—TNF-α, iNOS, NF-κB (p65), and NF-κB (p50) were retrieved from the RCSB Protein Data Bank (PDB IDs: 2TNF, 2ORR, 1MY5, and 8TKM, respectively) (Baeyens et al. 1999; Davey et al. 2007; Huxford et al. 2002; Zhu et al. 2023). Molecular docking calculations were conducted using Autodock Vina version 4.2.6 (Trott and Olson 2010). Throughout the docking procedure, Gasteiger partial charges were assigned to the bonds, and the electronegative differences between atoms and bonds were considered. A grid box was then created to cover the entire molecule to identify potential binding sites of FB_1_ with TNF-α, iNOS, NF-kB (p65), and NF-kB (p50). The grid box parameters were set as follows: [(TNF-α, centre x =  − 7.668 Å, y = 48.272 Å, and z =  − 47.205 Å and the dimensions were x = 68 Å, y = 66 Å, and z = 68 Å with an exhaustiveness of 10), (iNOS, centre x = 69.648 Å, y =  − 15.993 Å, and z = 50.142 Å and the dimensions were x = 50 Å, y = 68 Å, and z = 66 Å with an exhaustiveness of 10), (NF-κB (p65), centre x = 23.596 Å, y = 20.946 Å, and z = 38.586 Å and the dimensions were x = 72 Å, y = 80 Å, and z = 60 Å with an exhaustiveness of 10) and (NF-κB (p50), centre x =  − 52.442 Å, y = 16.741 Å, and z =  − 9.013 Å and the dimensions were x = 102 Å, y = 104 Å, and z = 116 Å with an exhaustiveness of 10)]. Docked conformations of the respective protein–ligand complexes were generated using the Lamarckian genetic algorithm and ranked in descending order according to their docking scores. These protein–ligand complexes were then visualized using UCSF Chimera to analyse key interactions between FB_1_ and the inflammatory proteins (Pettersen et al. 2004). The various binding conformations, along with their respective docking scores and root mean square deviations (RMSD), were calculated using Autodock Vina. The highest docking energy conformations for each interaction were analysed in this study.

### Materials

All reagents used in this study were of high grade and were sourced from reputable commercial suppliers. General laboratory reagents such as BSA, SDS, Tris, NaCl, KCl, glycerol and etc. were obtained from Sigma-Aldrich (St. Louis, Missouri, USA). Chemicals used for gene expression analysis, including gene specific primers (listed in Table 1), were synthesised by Inqaba Biotechnical Industries (Pretoria, South Africa) and other associated reagents were sourced from Thermo Fisher Scientific (Waltham, MA, USA). Materials required for protein extraction and Western blot analysis were generally obtained from Sigma-Aldrich (St. Louis, Missouri, USA) and Bio-Rad (Hercules, California, USA), while the utilised antibodies sources are detailed in Table 2. The Elisa kits were obtained from ABclonal (Woburn, Massachusetts, USA), with the exception of the DNA methylation kit, which was purchased from Abcam (Cambridge, United Kingdom). Nitric oxide assay reagents such as sulphanilamide and sodium nitrate were obtained from Sigma-Aldrich (St. Louis, Missouri, USA). Experimental treatment groups were prepared using FB_1_ obtained from Cayman Chemical (Michigan, USA). All the reagents listed in this section was prepared, optimised and sued according to the manufacturer’s instructions.

**Table 1:** The forward and reverse primer sequences with their correlating annealing temperatures used for qPCR (Inqaba Biotechnical Industries, Pretoria, South Africa)

Gene	Sequence (5′–3′)	Annealing temperature (°C)
*TNF-α*	Forward CATCTTCTCAAAATTCGAGTGACAA Reverse ACTTGGGCAGATTGACCTCAG	58
*NF-κB*	Forward ATGGCAGACGATGATCCCTAC Reverse CGGAATCGAAATCCCCTCTGTT	60
*IL-6*	Forward TCTATACCACTTCACAAGTCGGA Reverse GAATTGCCATTGCACAACTCTTT	58
*NLRP3 Inflammasome*	Forward ATCAACAGGCGAGACCTCTG Reverse GTCCTCCTGGCATACCATAGA	56
*IL-18*	Forward GTGAACCCCAGACCAGACTG Reverse CCTGGAACACGTTTCTGAAAGA	58
*Caspase 1*	Forward AATACAACCACTCGTACACGTC Reverse AGCTCCAACCCTCGGAGAAA	56
*IL-1β*	Forward TTCAGGCAGGCAGTATCACTC Reverse GAAGGTCCACGGGAAAGACAC	58
*GSDMD*	Forward CCATCGGCCTTTGAGAAAGTG Reverse ACACATGAATAACGGGGTTTCC	60
*Caspase 3*	Forward GGAGGCTGACTTCCTGTATGCTT Reverse CCTGTTAACGCGAGTGAGAATG	60
*CT-1*	Forward CTCCTCAATCTCATTCCTACCCC Reverse GCTGCACGTATTCCTCCAGAA	60
*IL-10*	Forward GCTCTTACTGACTGGCATGAG Reverse CGCAGCTCTAGGAGCATGTG	58
*MBD2*	Forward AGAACAAGGGTAAACCAGACCT Reverse ACTTCACCTTATTGCTCGGGT	58
*DNMT1*	Forward AGAGACCAGGATAAGAAACGCA Reverse CTCCTTTGATTTCCGCCTCAAT	60
*DNMT3A*	Forward GGCCGAATTGTGTCTTGGTG Reverse CCATCTCCGAACCACATGAC	60
*DNMT3B*	Forward AGCGGGTATGAGGAGTGCAT Reverse GGGAGCATCCTTCGTGTCTG	60
*GAPDH*	Forward AGGTCGGTGTGAACGGATTTG Reverse TGTAGACCATGTAGTTGAGGTCA

**Table 2:** Primary and secondary antibodies with their correlating catalogue numbers and dilutions used for western blot

Protein	Catalogue number	Dilution
P-NF-κB	Cell Signalling Technology, 3033S, Danvers, Massachusetts, USA	1:1000
Caspase 3	Cell Signalling Technology, 9665, Danvers, Massachusetts, USA	1:1000
DNMT1	Cell Signalling Technology, 5032S, Danvers, Massachusetts, USA	1:1000
MBD2	Abcam, ab188474,Cambridge, United Kingdom	1:1000
Goat anti-rabbit	Cell Signalling Technology, #7076P2, Danvers, Massachusetts, USA	1:5000
Goat anti-mouse	Cell Signalling Technology, #7076P2, Danvers, Massachusetts, USA	1:5000
β-actin	Sigma-Aldrich, A3854, St. Louis, Missouri, USA	1:5000

### Animal treatment

C57BL/6 male mice aged 6–8 weeks, were obtained from the Africa Health Research Institute at the University of KwaZulu-Natal, Durban, South Africa. The mice were housed in compliance with the ARRIVE guidelines and the regulations set forth by the University of KwaZulu-Natal Animal Research Ethics Committee (Ethics approval number: AREC/079/016). The mice weighed an average of 20 ± 2.99 g and were divided randomly into two groups containing 5 mice each of non-treated (control) and treated (FB_1_) mice. The mice were adapted under normal operating laboratory conditions (temperature = 25 °C, humidity = 40–60%, 12 h light/dark cycle). These mice were fed a mice pellet diet and normal drinking water for the duration of the experiment. FB1 (catalogue number: 62580;  ≥ 95% purity) was purchased from Cayman Chemicals (Michigan, US). To prepare the FB_1_ stock solution, 0.575 mg FB_1_ was weighed and dissolved in 1.25 mL of 0.1 M PBS which was prepared fresh everyday of administration, if not used it was stored at 4 °C for short term use. The working solution was regulated to deliver 5 mg/kg body weight based on the average mouse weight (20 ± 2.99 g). Therefore, the average administration volume was 0.25 mL per 23 g body weight, calculated to ensure accurate dosing. The mice were orally administered either with 0.1 M phosphate-buffered saline (PBS) (control group) or 5 mg/kg FB_1_ (FB_1_ group) at a rate of 0.25 ml/23 g once for a period of 24 h (Sibiya 2018; Ghazi et al. 2020). A 24-h exposure period was selected as this study was designed as a pilot study intended to establish a baseline response. Therefore, the primary objective was to assess whether an acute response of FB_1_ could initiate measurable biological effects such as inflammatory responses and epigenetic changes before progressing to longer-term chronic exposure models. Subsequently the acute exposure design was selected because it allowed for the identification of early signalling events and molecular alterations that underpin downstream effects. On a broader spectrum this approach therefore allows for critical preliminary data to justify subsequent investigations in chronic exposure scenarios. Thereafter, the mice were euthanised using halothane anaesthesia and the hearts were harvested by a qualified veterinary surgeon. The hearts were washed three times in 0.1 M PBS and stored in Cytobuster reagent (500 μl; Novagen, CA, USA), Qiazol reagent (500 μl; Qiagen, 79,306) and 0.1 M PBS for protein, RNA and homogenate extraction, respectively.

### Tissue preparation

Whole mouse hearts stored in PBS, Cytobuster, and Qiazol were thawed on ice and homogenised. Mice heart tissue (approximately 150 mg) was homogenised in 1.5 mL PBS/Qiazol/Cytobuster (1:10) were homogenised using a homogenizer, as per standard protocols. The homogenates were transferred to microcentrifuge tubes and subjected to centrifugation at 10,000 × g for 10 min at 4 °C. The supernatants were collected for subsequent DNA and RNA isolation, homogenate, and protein analyses. These samples were then utilised for quantitative polymerase chain reaction (qPCR), nitric oxide synthase (NOS) assay, enzyme-linked immunosorbent assay (ELISA), and Western blotting.

### Quantitative polymerase chain reaction (qPCR)

The mice tissue stored in Qiazol reagent underwent the specified tissue preparation procedure for total RNA extraction. After removal from the freezer, the samples were thawed and supplemented with 100 µl of chloroform. They were then vortexed for 15 s and incubated at room temperature for 2–3 min. Following this, the samples were centrifuged at 12,000 × g for 15 min at 4 °C. The resulting aqueous phase was carefully transferred into clean microcentrifuge tubes. To the supernatant, 250 µl of isopropanol was added and gently mixed before freezing at − 80 °C. After an overnight freeze, the samples were thawed on ice and centrifuged at 12,000 × g at 4 °C for 20 min. The supernatant was discarded, and the pellet was washed with 500 µl of 75% ethanol. While in ethanol, the samples were centrifuged at 7400 × g at 4 °C for 15 min. After centrifugation, the ethanol was removed, and the pellets were air-dried for 45 min. The dried pellets were then resuspended in 15 µl of nuclease-free water and allowed to equilibrate at room temperature for about 3 min. The extracted RNA was measured, and its quality was assessed using the A260/A280 ratio with a Nanodrop 2000 spectrophotometer (Thermo-Fisher Scientific, Waltham, MA, USA). Finally, all RNA samples were standardised to a concentration of 500 ng/µl.

Thereafter 1 µl of standardised RNA samples were utilised for cDNA synthesis, adhering to the instructions provided by the cDNA Synthesis Kit (K1652, Thermo-Fisher Scientific, Waltham, MA, USA). Following the manufacturer’s guidelines, the expression of relevant inflammatory and DNA methylation genes (Table 1) was assessed using SYBR® Green Supermix (A25742, Thermo-Fisher Scientific, Waltham, MA, USA) and the QuantStudio™ 3 Real-Time PCR Software, Version 1 (Waltham, MA, USA). The thermocycling protocol for each gene included an initial denaturation at 95 °C for 8 min, followed by 40 cycles of 15 s at 95 °C. The annealing stage was performed at the temperatures specified in Table 1 for 40 s, followed by a final extension at 72 °C for 30 s. Gene expression was normali*s*ed using GAPDH, a housekeeping gene uniformly expressed across all cells and tissues. The data were analysed using the comparative threshold cycle (Ct) method and expressed as relative fold changes compared to the control.

### Western blotting

To quantify the protein expression of key inflammatory factors and mediators phosphorylated-NF-κB (P-NF-κB) and caspase 3 as well as DNA methylation-associated factors (Methyl-CpG-binding domain protein 2) MBD2 and DNMT1, western blotting was employed. Proteins were extracted from untreated (control) and FB_1_-treated mice using Cytobuster (200 µl; Novagen, 71,009, Darmstadt, Germany), supplemented with protease and phosphatase inhibitors (Roche; 05892791001 and 04906837001, respectively, Basel, Switzerland.). Following tissue homogenisation (as described in the tissue preparation section), samples were centrifuged, and the resulting homogenate was used for protein analysis. Protein concentrations were determined using the (Bicinchoninic acid assay) BCA assay and standardised to 2 mg/ml. These standardised protein samples were mixed with 1X Laemmli buffer [dH2O, 0.5 M Tris–HCl (pH 6.8), glycerol, 10% SDS, 5% β-mercaptoethanol, 1% bromophenol blue] in a 1:1 ratio and boiled at 100 °C for 5 min to denature the proteins. For Western blotting, 30 µl of total protein was loaded per lane, this amount was standardised across all samples to ensure consistency and reliable detection of target proteins. Proteins were separated by size using sodium dodecyl sulphate polyacrylamide gel electrophoresis, with a 10% resolving gel and a 4% stacking gel, at 150 V for 1 h. Following electrophoresis, proteins were transferred to a nitrocellulose membrane at 20 V for 30 min using the Bio-Rad Trans-Blot® Turbo Transfer System (Hercules, California, USA). The membrane was blocked with 5% BSA in Tris-buffered saline containing 0.05% Tween 20 [TTBS; 150 mM NaCl, 3 mM KCl, 25 mM Tris, 0.05% Tween 20, dH2O, pH 7.5 (St. Louis, Missouri, USA.)] for 1 h at room temperature. Blocked membranes were incubated overnight at 4 °C with primary antibodies (Table 2). The following day, membranes were washed five times and incubated with HRP-conjugated secondary antibodies (Table 2, anti-rabbit and anti-mouse) for 1 h at room temperature. After incubation, membranes were washed and developed using the Clarity™ Western ECL Substrate Kit (Bio-Rad, #170-5060, Hercules, California, USA). Protein band images were captured using the Invitrogen iBright CL1500 Imaging System (Thermo-Fisher Scientific, Waltham, MA, USA). Membranes were thereafter stripped for probing with the housekeeping protein β-actin by incubating them with hydrogen peroxide for 30 min at 37 °C, followed by a TTBS wash for 10 min. Membranes were then probed with anti-β-actin (Table 2) for 30 min at room temperature. Protein bands were analysed using the iBright Imaging System software V1 and expressed as relative band density (RBD). This intensity was normalised using the intensity of the β-actin bands, ensuring accurate and reliable quantification of protein expression levels.

### Nitric oxide synthase (NOS) assay

The NOS assay was utilised to quantify NO concentrations in untreated and FB_1_-treated mice. Sodium nitrate standards (0, 25, 50, 75, 100, 125, 150, 175, 200 µM) and mouse heart homogenate were prepared by homogenising heart tissue (approximately 150 mg) was homogenised in 1.5 mL PBS (1:10) using a homogeniser, as per standard protocols. Thereafter 50 µl aliquots of the sample was plated in triplicate into a 96-well plate. Subsequently, 50 µl of VCl3, 25 µl of sulphanilamide, and 50 µl of (N-(1-Naphthyl) ethylenediamine dihydrochloride) NEDD were rapidly added to the wells containing the samples (St. Louis, Missouri, USA.). The plate was then incubated at 37 °C with 5% CO_2_ for 45 min in the dark, as the assay is light sensitive. Following incubation, absorbance was measured using the SPECTROstar® nano-microplate reader at wavelengths of 540 nm and 690 nm. The obtained absorbance values were averaged and used to generate a standard curve. The standard curve equation was then employed to calculate the concentrations of nitrate/nitrite present in the control and FB_1_-treated mouse heart homogenate.

### Enzyme-linked immunosorbent assay (ELISA)

#### Cytokine ELISA

The levels of proinflammatory cytokines TNF-α (RK00027, Abclonal), IL-6 (RK00008, Abclonal), IL-1β (RK00006, Abclonal), IL-10 (RK00016, Abclonal), and TGF-β1 (RK00057, Abclonal) (Woburn, Massachusetts, USA) were quantified using ELISA kits. Biological samples for these assays were prepared according to the previously described tissue preparation steps, specifically utilising homogenate samples. Prior to the experiment, all reagents were equilibrated to room temperature. Following the manufacturer’s instructions, the reagents were prepared as follows: the standard solution was created by reconstituting the lyophilised standard with 1 ml of standard sample diluent (R1), yielding a stock solution of 2000 pg/ml. Serial dilutions ranging from 2000 to 0 pg/ml were then prepared using 250 µl of R1. The working biotin-conjugate antibody was prepared by diluting 20 µl of concentrated biotin conjugate into 1980 µl of R2, creating 2000 µl of working biotin solution. The working streptavidin-HRP solution was prepared by adding 20 µl of concentrated streptavidin-HRP to 1980 µl of streptavidin-HRP diluent (R3), resulting in 2000 µl of working streptavidin-HRP buffer. The wash buffer was prepared by diluting 20 ml of concentrated wash buffer in 380 ml of deionised water to make 400 ml of solution. The ELISA procedure commenced with washing the wells three times with 350 µl of wash buffer solution. After washing, 50 µl of standards, controls, and FB_1_ samples were aliquoted into the wells and incubated for 2 h at 37 °C. Following incubation, the solutions were aspirated, and the washing steps were repeated. Subsequently, 50 µl of the working biotin-conjugate antibody was added to each well and incubated at 37 °C for 1 h. After incubation, the wells were aspirated, and three additional washing steps were performed. Then, 50 µl of working streptavidin-HRP conjugate was added and incubated for 30 min at 37 °C. Following this, the wells were aspirated, and the washing steps were repeated. Once washing was completed, 50 µl of (3,3′,5,5′-Tetramethylbenzidine) TMB substrate was added to each well and incubated in the dark for 15–20 min at 37 °C. After incubation, 25 µl of stop solution was added, and optical density was measured within 5 min using the SPECTROstar® Nano microplate reader at 450 nm with a reference wavelength of 630 nm. The data obtained were used to generate a standard curve, and the equation from the curve was extrapolated to quantify the concentrations of the relevant cytokines.

#### Global DNA methylation ELISA

Heart tissue was homogenised in Qiazol reagent at a ratio of 1:10 (approximately 150 mg tissue in 1.5 mL Qiazol). The homogenate was centrifuged to remove non-homogenised pieces and debris. RNA was then extracted from the aqueous phase of the homogenate using Qiazol. Following RNA isolation, the interphase and lower phenol–chloroform phase remaining after removal of the aqueous phase were retained for DNA isolation. This DNA was subsequently used for the DNA methylation ELISA analysis. To the isolate, 30 µl of 100% ethanol was added per 1 ml of Qiazol, and the mixture was gently inverted for 2–3 min. This was followed by centrifugation at 2000 × g for 5 min at 4 °C to pellet the DNA. The DNA pellet was resuspended in 300 µl of 0.1 M sodium citrate and incubated for 30 min with occasional inversion. The sample was then centrifuged at 2000 × g for 5 min at 4 °C. The pellet was resuspended in 1 ml of 75% ethanol, incubated for 10–20 min with gentle inversion, and centrifuged at 2000 × g for 5 min at 4 °C. After centrifugation, the supernatant was discarded, and the pellet was air-dried for 10 min. The pellet was then solubilised in 300 µl of 8 mM sodium hydroxide, followed by centrifugation at 2000 × g for 10 min at 4 °C. The DNA yield was measured and standardised to a concentration of 15 ng/µl. The standardised DNA was used to quantify global DNA methylation using the Methylated DNA Quantification ELISA Kit (Abcam, ab117128, Cambridge, United Kingdom) according to manufacturer’s instructions. The plate was then read using the SPECTROstar® Nano microplate reader at 450 nm. The optical densities obtained were used to generate a standard curve, which was employed to determine the levels of 5-methylcytosine (5-mC) present.

### Statistical analysis

Statistical analyses were conducted using GraphPad Prism. Each assay was evaluated using a t-test with Welch’s correction. Relative changes in gene expression were assessed using the comparative threshold cycle method. Standard curves for the NOS assay, BCA assay, and all ELISAs were prepared using Excel to determine the concentrations of relevant cytokines and DNA methylation levels. To ensure reliability, each assay was performed in triplicate. Laboratory data were presented as mean ± standard error of the mean (SEM), with significance levels indicated by p-values: *p < 0.05, **p < 0.005, and ***p < 0.0001.

## Results

### FB_1_ interacts with TNF-α, iNOS, NF-κB (p65), and NF-κB (p50)

**Fig. 1:**
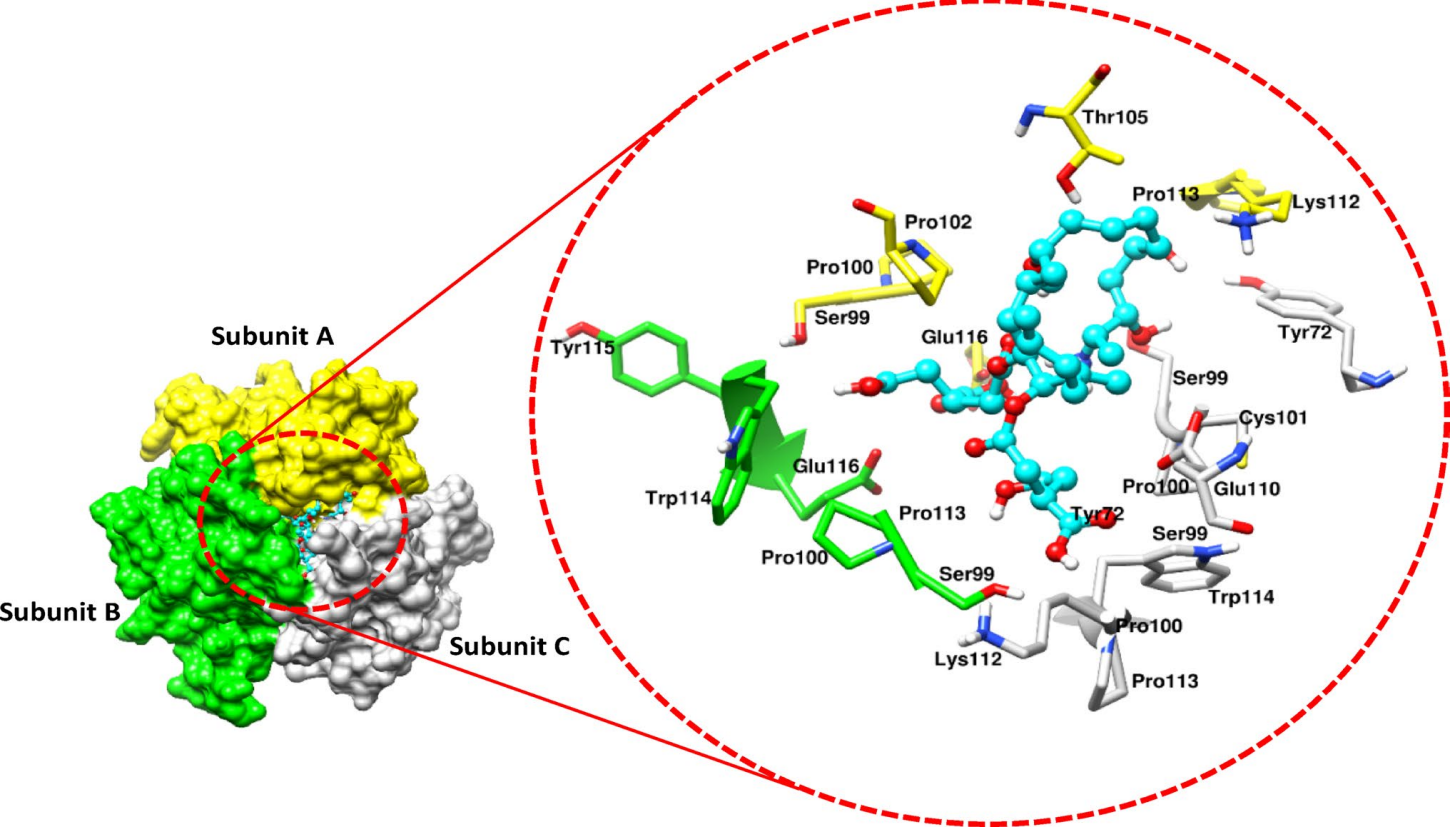
The diagram depicts the docking of FB_1_ between subunits A, B, and C, potentially leading to the activation of TNF-α. The TNF-α protein is presented in a surface view, with subunits A, B, and C coloured yellow, green, and white, respectively. FB_1_ is illustrated as a cyan blue stick structure. A topographical view highlights FB_1_’s most stable binding within the trimeric channel of TNF-α, with Tyr115 and Pro113 key residues involved in TNF-α function highlighted in green. UCSF Chimera was used for visualisation and image preparation

**Fig. 2:**
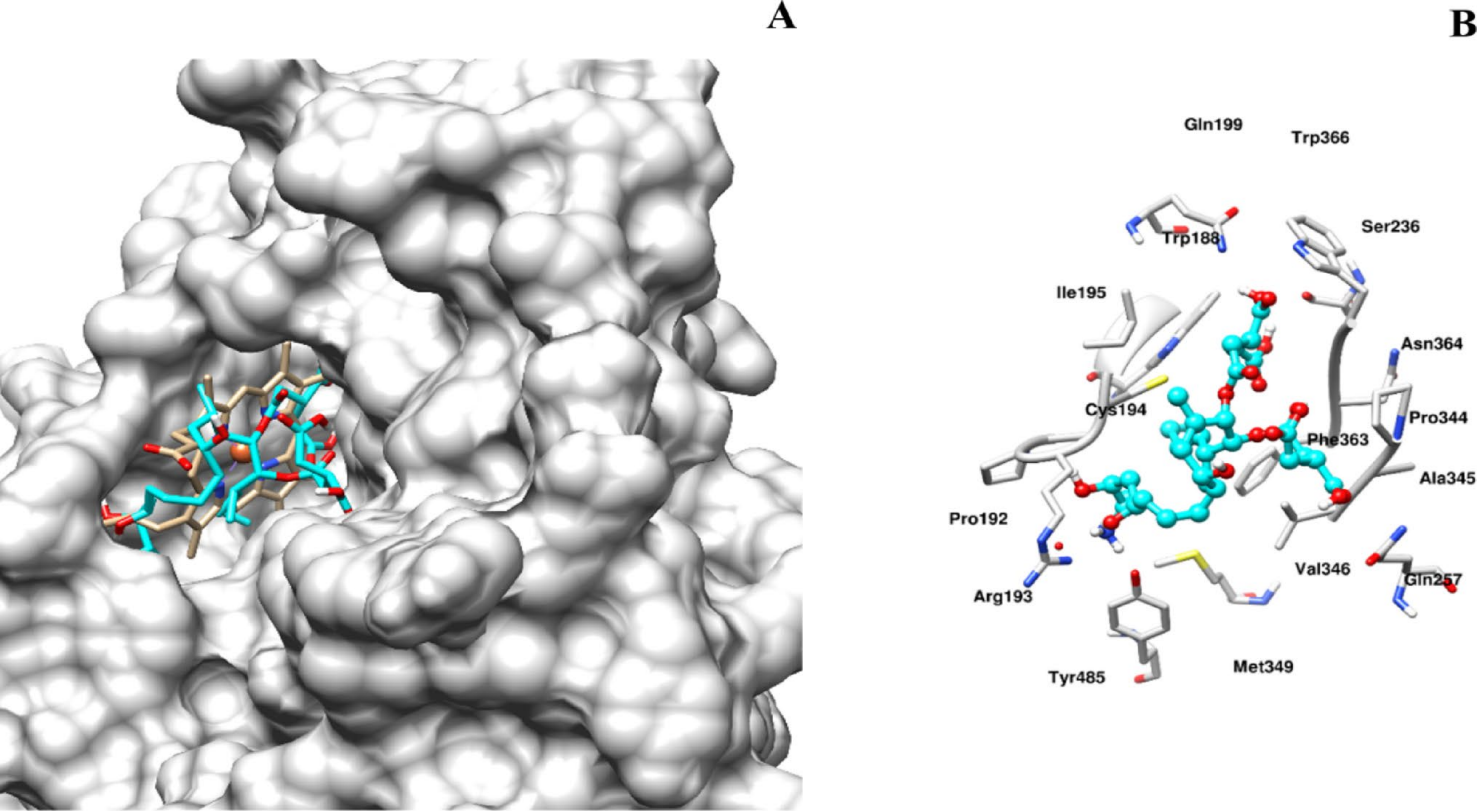
The figure illustrates the interaction of FB_1_ with the heme molecule of iNOS, highlighting the selective binding of FB_1_ to a pocket around the heme group, which is characteristic of the iNOS molecule. FB_1_ potentially interacts with crucial residues, including Phe363, Cys194, and Tyr485, which are involved in iNOS enzyme function. UCSF Chimera was used for visualisation and image preparation

The molecular docking of FB_1_ was investigated on four inflammation-related proteins: TNF-α, iNOS, NF-κB (p65), and NF-κB (p50), to identify potential interactions. The docking energies for the nine conformations of FB_1_ with TNF-α ranged from − 5.4 to − 4.8 kcal/mol, with the most stable conformation inserting itself in the cleft between subunits A, B, and C (Fig. 1). Conversely, FB_1_ demonstrated a higher docking affinity for iNOS, with scores ranging from − 6.5 to − 5.6 kcal/mol, with the most stable conformation binding near the heme binding site on the surface of iNOS (Fig. 2A). Additionally, a possible interaction with the cysteine 194 residue was observed (Fig. 2B).

**Fig. 3:**
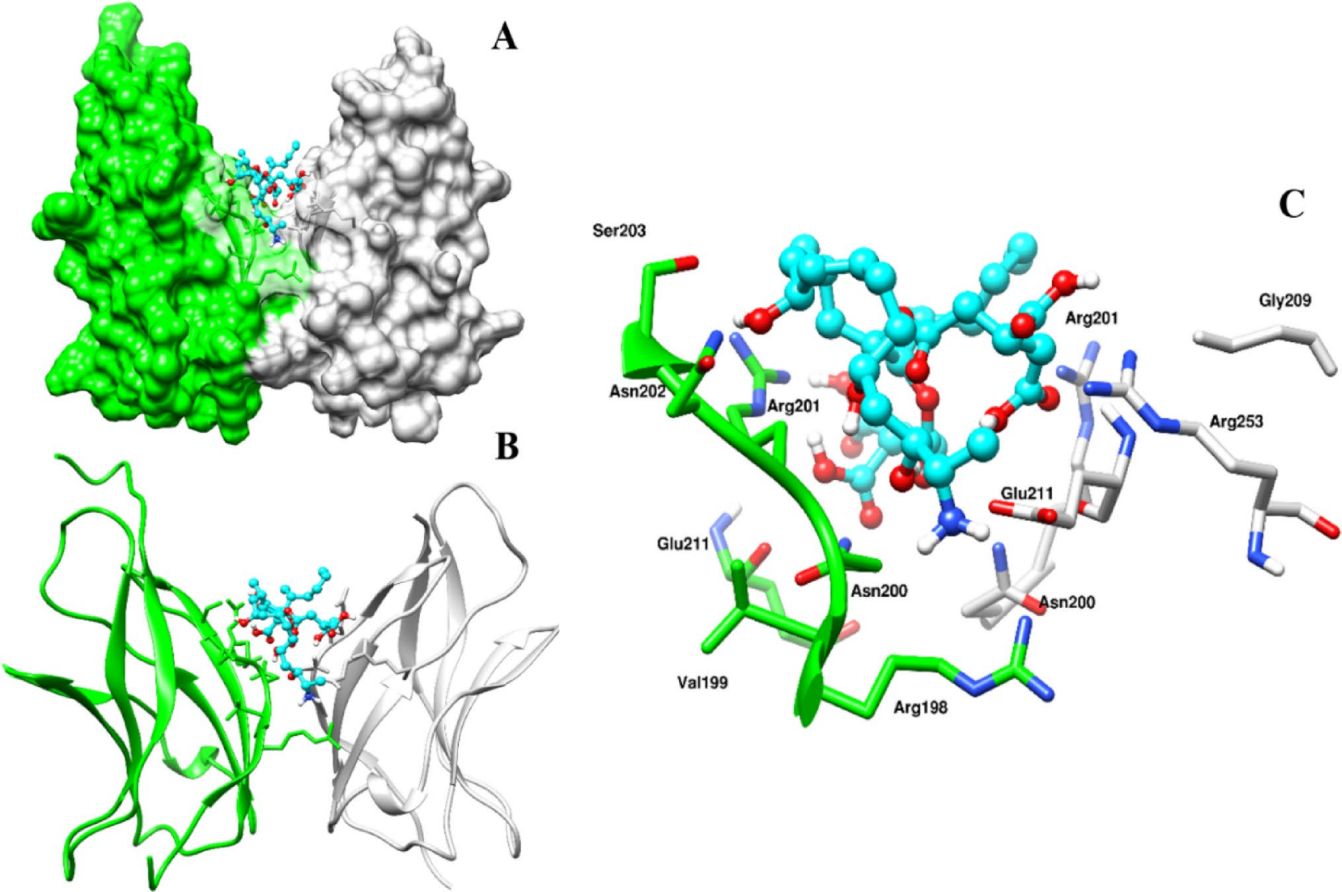
The interaction of FB_1_ with NF-κB (p65) subunits is depicted in several views to highlight potential binding sites and interactions. Picture **A** presents a surface view of NF-κB (p65), with its two subunits shown in white and green, while FB_1_ is represented as a cyan blue stick figure, emphasizing possible key interactions between FB1 and NF-κB (p65). Picture **B** offers a ribbon rendition of Picture **A**, providing an alternative perspective on the structure. Picture **C** illustrates a stick figure representation, detailing the key residues that FB_1_ could potentially interact with. The diagram was prepared using UCSF Chimera

**Fig. 4:**
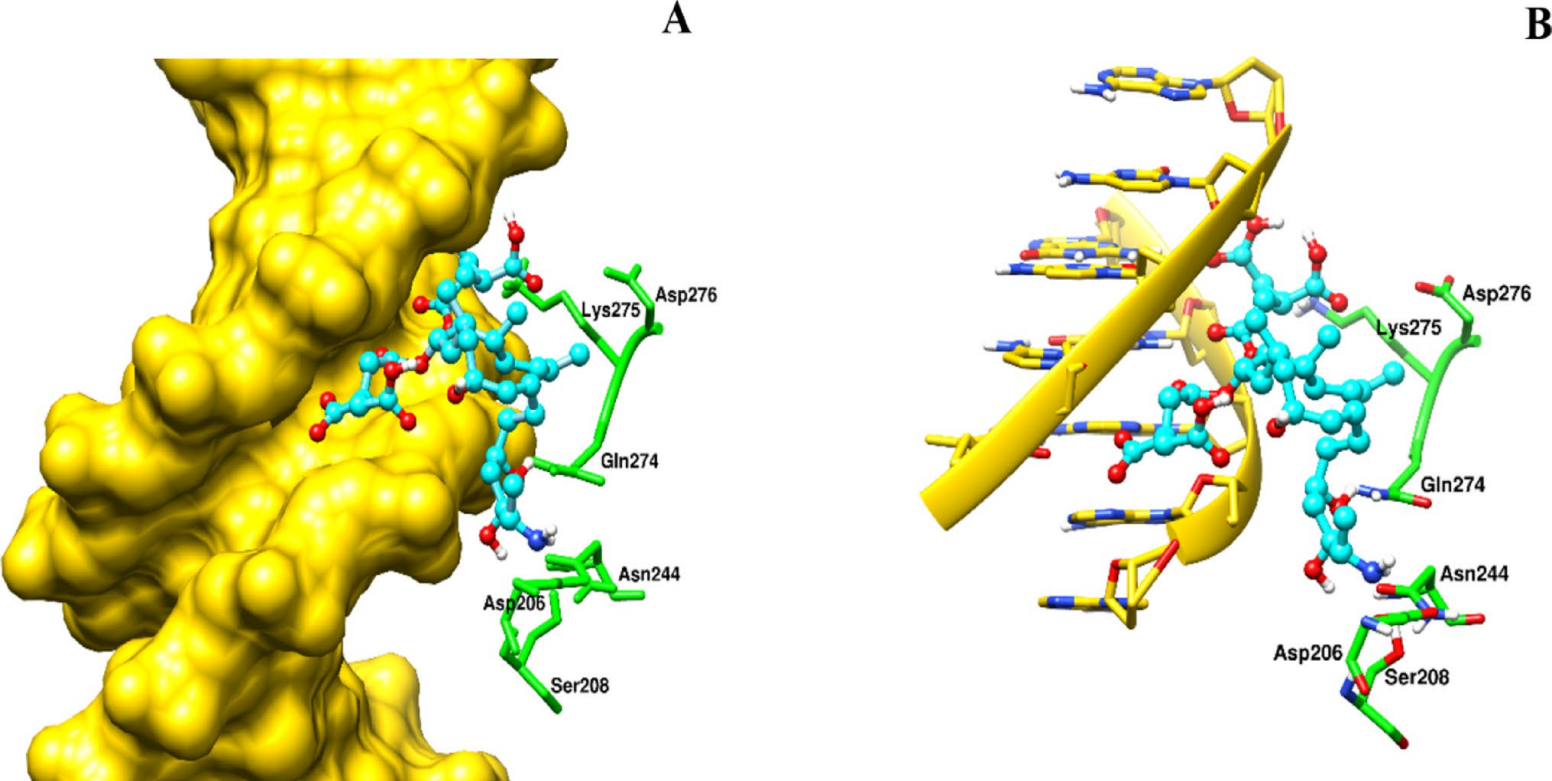
The potential interaction of FB_1_ with DNA and the NF-κB (p50) protein subunit is depicted, suggesting that this interaction could lead to altered transcriptional activity of NF-κB (p50). Picture **A** shows the surface structure of the DNA within the NF-κB (p50) complex, with FB_1_ and the protein subunit represented as stick figures in blue and green, respectively. Picture **B** provides a stick figure depiction of the DNA, FB_1_, and NF-κB (p50), highlighted in yellow, green, and blue. UCSF Chimera was used for visualisation and image preparation

FB_1_’s docking energy with NF-κB (p65) was lower, ranging from − 4.8 to − 4.6 kcal/mol. The most stable conformation placed itself in the posterior region between the two subunits of NF-κB (p65), with Asn202 and Ser203 being critical residues for possible interaction with FB_1_ (Fig. 3A–C). The docking affinity for NF-κB (p50) was similar to that of iNOS, with values ranging from − 6.3 to − 5.8 kcal/mol. Notably, the stable binding of FB_1_ facilitated potential interactions between the DNA structure and the protein subunit of NF-κB (p50) (Fig. 4A). Key residues highlighted involved in the possible interaction with FB_1_ included Gln274, Asn244, and Lys275 (Fig. 4B).

### FB_1_ altered the gene expression of key inflammatory cytokines TNF-α, NF-κB, IL-6, NLRP3, and IL-18

**Fig. 5:**
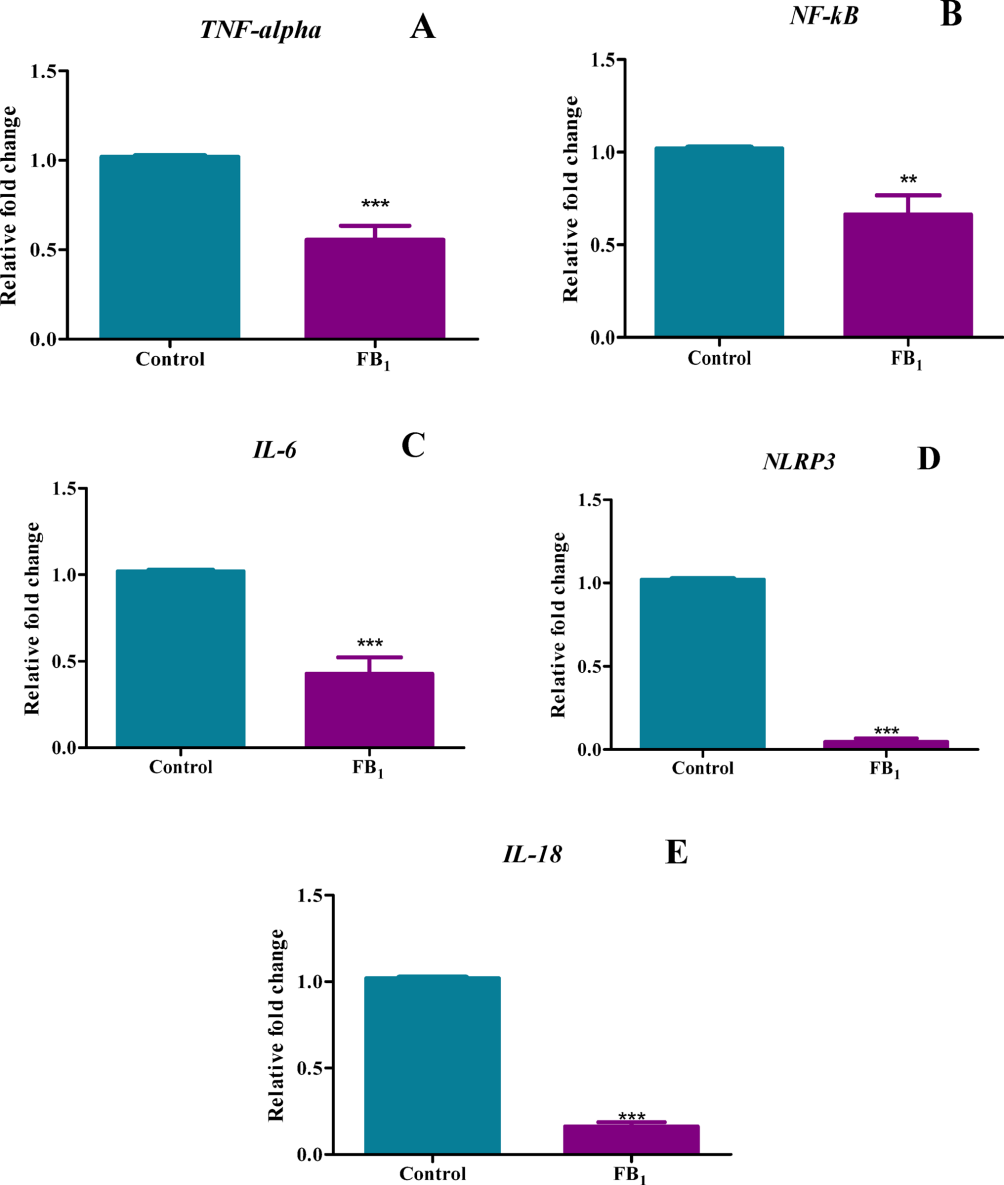
Treatment with FB_1_ downregulated several inflammatory genes. This figure illustrates the effect of FB_1_ on the gene expression of pro-inflammatory genes in C57BL/6 mice hearts. Treatment with FB_1_ resulted in a significant decline in the expression of several inflammatory genes, including TNF-α (**A**), NF-κB (**B**), IL-6 (**C**), NLRP3 (**D**), and IL-18 (**E**). The results are shown as mean ± SEM (*n* = 5/group). An unpaired t-test with Welch’s correction was used to determine statistical significance (**p < 0.005; ***p < 0.0001)

A key inflammatory cytokine during the initiation of a pro-inflammatory event is TNF-α. FB_1_ significantly reduced TNF*-α* expression with a percentage decrease of 44.49% (*p* < 0.0001) (Fig. 5A). The activation of NF-κB is driven by TNF-α induction; thus, this decrease in TNF-α corresponded with a significant reduction in *NF-κB* expression with a percentage decrease of 33.74% (*p* = 0.0041) (Fig. 5B). NF-κB activates downstream cytokines and factors, such as IL-6 and the NLRP3 inflammasome. FB_1_ induced a marked decline in the expression of *IL-6* represented by a percentage decrease of 57.22% (*p* < 0.0001) and *NLRP3* depicted a percentage decrease of 95.42%b (*p* < 0.0001) (Fig. 5C, D). Upon NLRP3 activation, IL-18 is typically activated from its dormant state; however, FB_1_ significantly decreased *IL-18* expression with a percentage decrease of 83.84% (*p* < 0.0001) (Fig. 5E), which corresponds to the reduction in *NLRP3* expression.

### FB_1_ downregulated the expression of inflammatory factors, caspases, and associated cell death genes including caspase-1, IL-1β, GSDMD, and caspase-3

**Fig. 6:**
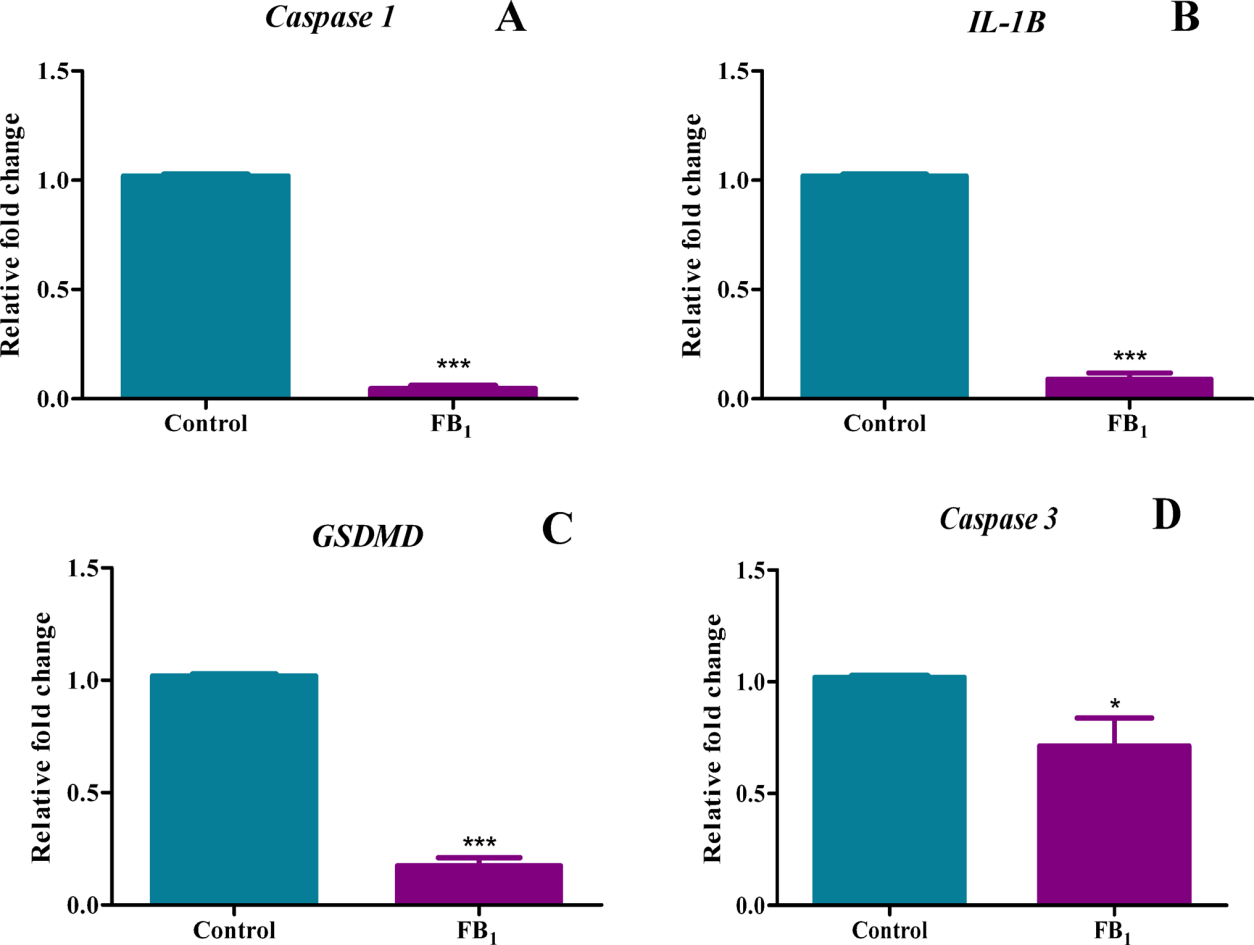
A decrease in gene expression of inflammatory factors associated with cell death proteins was induced by FB_1_. The effect of FB_1_ on the gene expression of pro-inflammatory genes and cell death proteins in C57BL/6 mice hearts. A decrease in gene expression of inflammatory factors associated with cell death proteins was induced by FB_1_. A significant decline in caspase 1 (**A**), IL-1β (**B**), GSDMD (**C**), and caspase 3 (**D**) gene expression was observed in mice treated with FB1. The results are shown as mean ± SEM (*n* = 5/group). An unpaired t-test with Welch’s correction was used to determine statistical significance (*p < 0.05; ***p < 0.0001)

The activation of the NLRP3 inflammasome is responsible for the activation of caspase-1, which subsequently cleaves pro-IL-1β into its active form, IL-1β. FB_1_-treated mice exhibited a significant reduction in the gene expression of both *caspase-1* represented by a percentage decrease of 95.28% and *IL-1β* depicted by a percentage decrease of 91.01% (*p* < 0.0001) (Fig. 6A, B, respectively), which corresponded with the observed changes in the gene expression of upstream inflammatory factors. Caspase-1 activation is not limited to cytokine activation but also involves the executioner of pyroptosis, GSDMD. FB_1_ significantly decreased expression of *GSDMD* with a percentage decrease of 82.61% (*p* < 0.0001) (Fig. 6C). The pyroptotic pathway is also influenced by another member of the Gasdermin family, GSDME, which is activated by caspase-3. FB_1_ induced a significant decline in the expression of *caspase-3* defined by a percentage decrease of 28.68% (*p* = 0.0283) (Fig. 6D), indicating an impediment of the alternative pyroptotic pathway, consistent with the aforementioned findings.

### Decreased gene expression of key regulatory factors in the inflammation pathway, including CT-1 and IL-10, induced by FB_1_

**Fig. 7:**
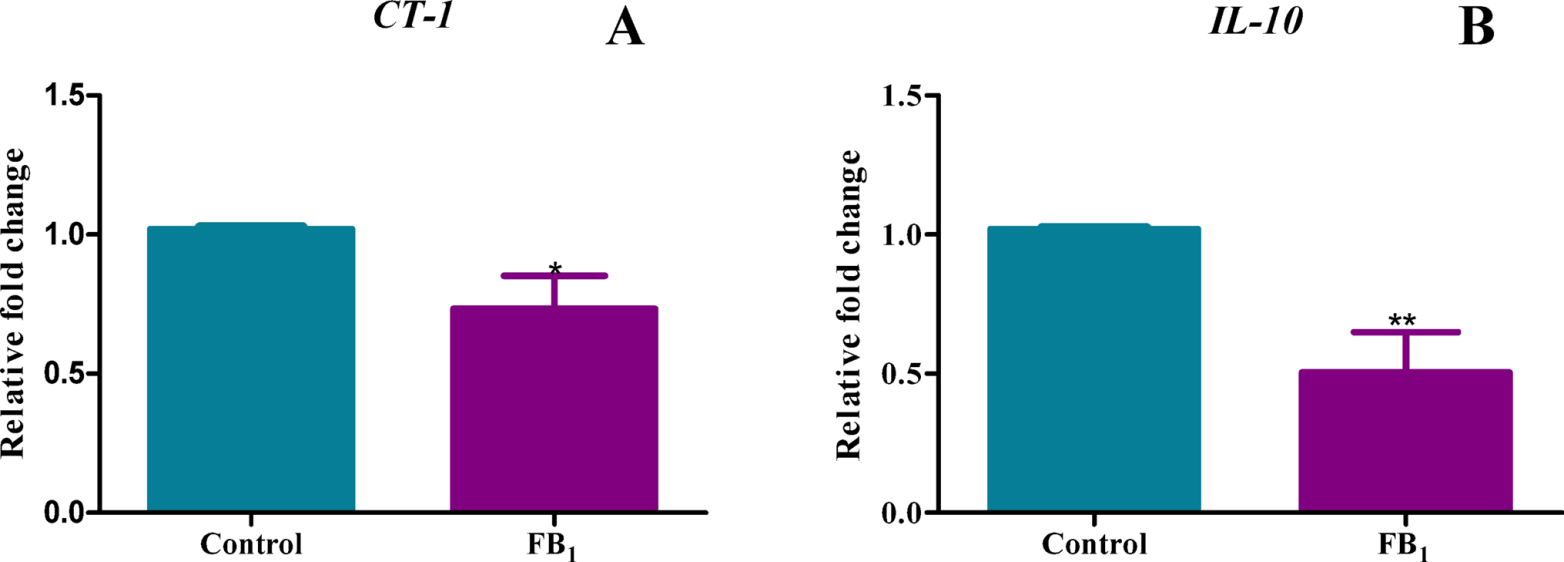
A decrease in gene expression of associated inflammatory factors, CT-1 and IL-10 was induced by FB_1_. The effect of FB_1_ on the gene expression of pro-inflammatory genes in C57BL/6 mice hearts. A decrease in gene expression of associated inflammatory factors, CT-1 and IL-10, was induced by FB_1_. A significant decrease in chemokine CT-1 (**A**) and anti-inflammatory cytokine IL-10 (**B**) was observed after acute 24-h exposure to FB_1_. The results are shown as mean ± SEM (n = 5/group). An unpaired t-test with Welch’s correction was used to determine statistical significance (*p < 0.05; **p < 0.005)

An emerging chemokine involved in the inflammatory response is CT-1, which has been shown to activate NF-κB through its interaction with the IκBα molecule. FB_1_ downregulated the gene expression of CT-1 with a percentage decrease of 26.67% (*p* = 0.0301) (Fig. 7A), suggesting that its downstream effects, such as NF-κB activation, are inhibited. To counteract the pro-inflammatory response, IL-10 is typically activated to limit inflammation and promote healing. However, FB₁ also decreased IL-10 gene expression represented by a percentage decrease of 49.58% (*p* = 0.0031) (Fig. 7B), indicating a more complex interplay in FB_1_'s ability to modulate gene expression.

### Acute exposure to FB_1_ leads to an increase in reactive nitrogen metabolites

**Fig. 8:**
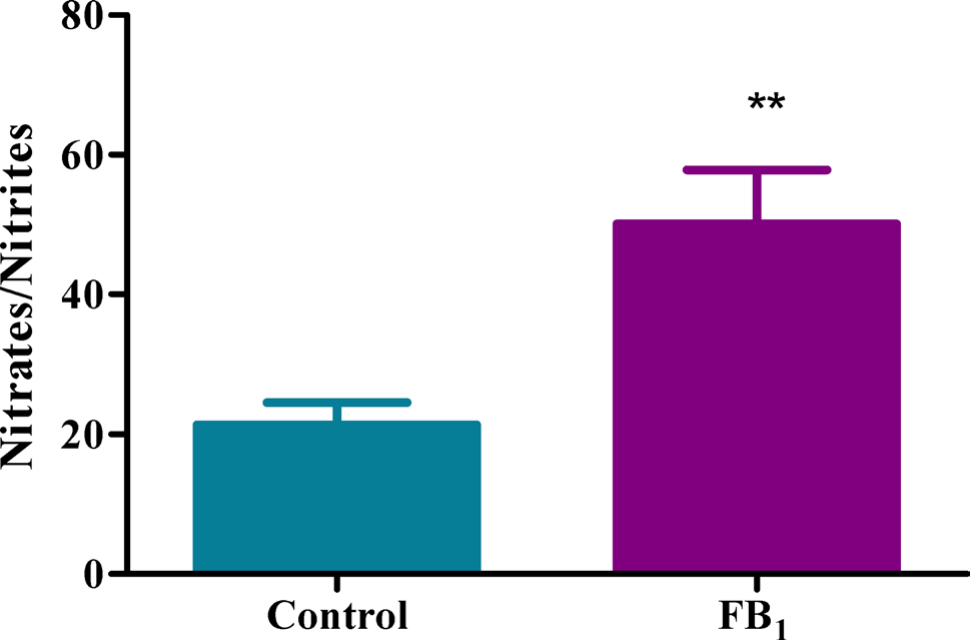
FB_1_ induced Nitrate/Nitrite concentration during acute exposure. The effect of FB_1_ on the gene expression of pro-inflammatory genes in C57BL/6 mice hearts. FB_1_ treatment resulted in a significant upregulation of RNS, contributing to the inflammatory response. A significant increase in nitrate/nitrite concentration was observed during acute exposure to FB_1_ (**p < 0.005). The results are shown as mean ± SEM (*n* = 5/group). An unpaired t-test with Welch’s correction was used to determine statistical significance (**p < 0.005)

A vital modulator of the inflammatory response is NO; however, due to its short half-life, its precursor metabolites, NO_2_ and NO_3_, can provide insight via the NOS assay. In this study, acute exposure of FB_1_ revealed an upregulation in RNS concentration with a fold increase of 2.35 (*p* = 0.0028) (Fig. 8), which can lead to alterations in the inflammatory response.

### Increased protein expression of mediators of the inflammatory response, including P-NF-κB and caspase-3, triggered by FB_1_ treatment

**Fig. 9:**
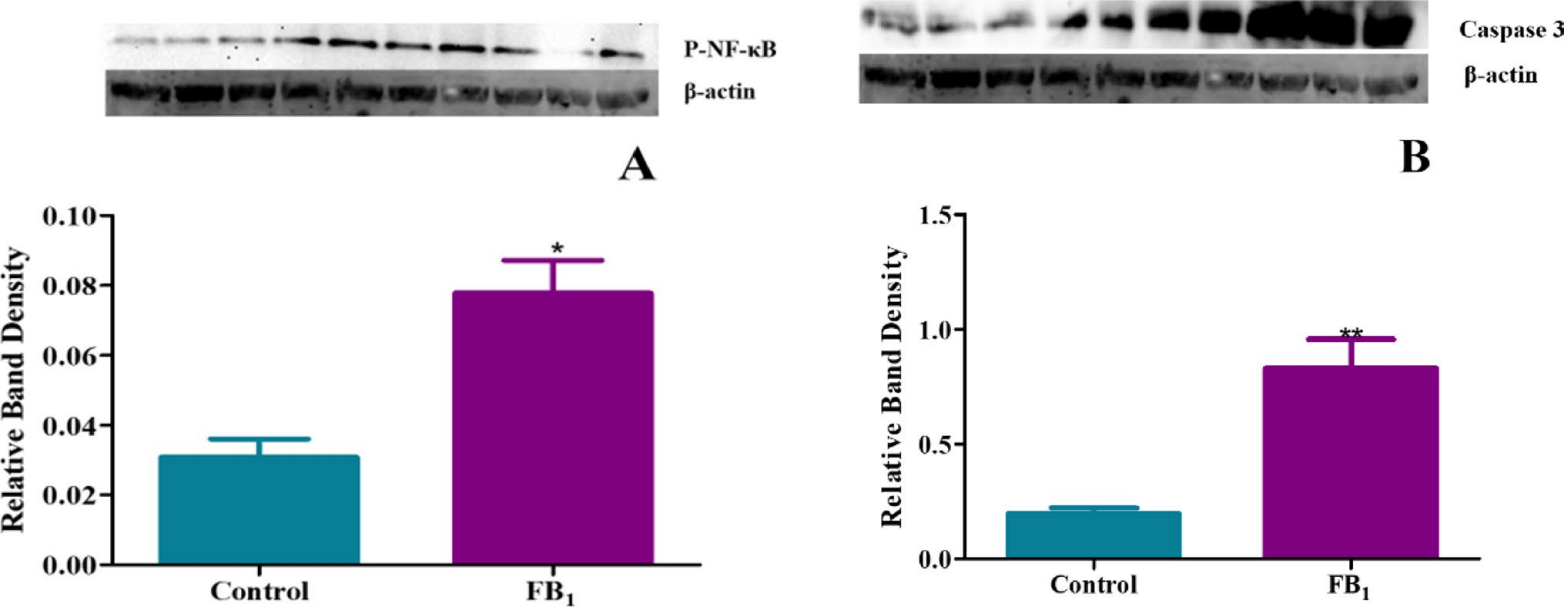
The treatment with FB_1_ led to upregulation in proinflammatory cytokine expression The effect of FB_1_ on the protein expression of pro-inflammatory proteins in C57BL/6 mice hearts. The transcription regulator of cytokine production in its active form showed significantly increased P-NFKB expression (**A**) and Caspase 3 (**B**). Bands 1 to 5 represent the control, and bands 6 to 10 represent the FB_1_ treatment. The results are shown as mean ± SEM (*n* = 5/group). An unpaired t-test with Welch’s correction was used to determine statistical significance (*p < 0.05; **p < 0.005)

A key transcriptional activator of crucial pro-inflammatory cytokines that heightens the pro-inflammatory response is NF-κB. When NF-κB becomes activated through phosphorylation, it can lead to the transcriptional activation of downstream mediators of the inflammatory process. In this study, phosphorylated NF-κB (P-NF-κB) was significantly upregulated by FB_1_, indicating an increase in subsequent pro-inflammatory cytokines showed by a fold increase of 2.52 (*p* = 0.0051) (Fig. 9A). The exacerbation of the inflammatory response has been shown to activate cell death pathways. In this investigation, executioner caspase-3 was significantly increased by a fold change of 4.22 by FB_1_, indicating the onset of programmed cell death (apoptosis) (*p* = 0.0082) (Fig. 9B).

### Treatment with FB_1_ led to significant upregulation of inflammatory cytokines TNF-α, IL-6, IL-1β, IL-10, and TGF-β1

**Fig. 10:**
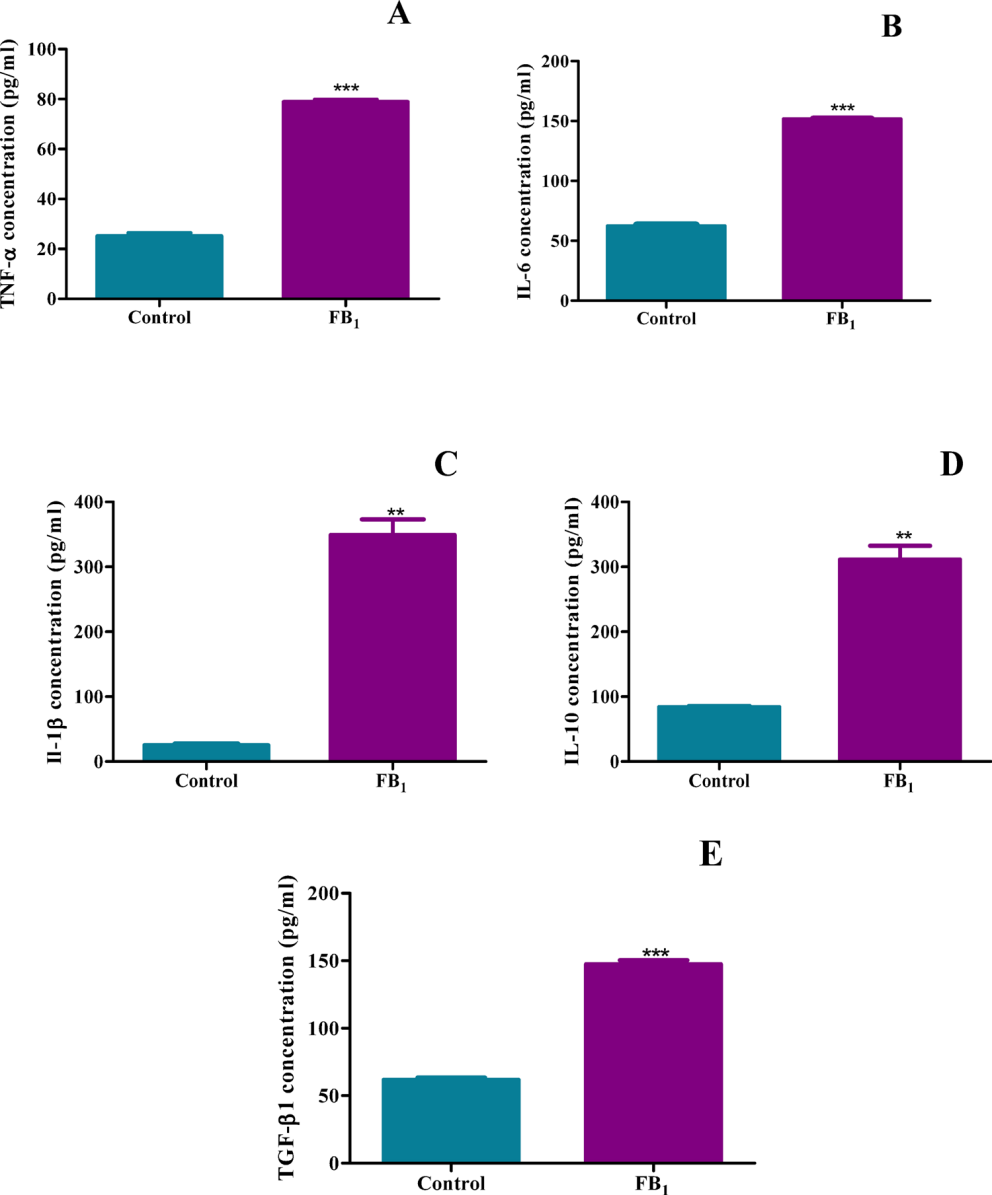
The induction of inflammatory factors by FB_1_ in mice. The effect of FB_1_ on the protein expression of pro-inflammatory proteins in C57BL/6 mice hearts. Homogenatelevels of TNF-α (**A**), IL-6 (**B**), IL-1β (**C**), IL-10 (**D**), and TGF-β1 (**E**) were significantly upregulated in mice after FB1 exposure. The results are shown as mean ± SEM (*n* = 5/group). An unpaired t-test with Welch’s correction was used to determine statistical significance (*p < 0.05; **p < 0.005; ***p < 0.0001)

The quantification of inflammatory cytokines in the homogenate revealed a significant contrast to the expression of inflammatory genes. TNF-α, typically produced by macrophages in response to toxic insults, was significantly increased by FB_1_ depicted by a fold increase of 3.13 (*p* < 0.0001) (Fig. 10A). Both IL-6 and IL-1β were highly expressed with IL-6 exhibited by a fold increase of 2.44 whereas IL-1β showcased a fold change of 13.89 (*p* < 0.0001) (Fig. 10B, C) due to FB_1_, indicating an increase in the pro-inflammatory response. Conversely, the anti-inflammatory cytokine IL-10 showed a concurrent increase in response to FB_1_ exposure with a fold change of 3.71 (*p* = 0.0087) (Fig. 10D). Furthermore, TGF-β1, a cytokine known for its involvement in inflammation and its association with cardiac pathology such as heart failure and cardiac fibrosis, was significantly upregulated as depicted by a fold increase of 2.39 (*p* < 0.0001) (Fig. 10E). These data highlight the potential of FB_1_ to trigger pathways associated with cardiac distress.

### Altered expression of DNMT1 and MBD2 proteins and increased global DNA methylation levels following FB_1_ treatment

**Fig. 11:**
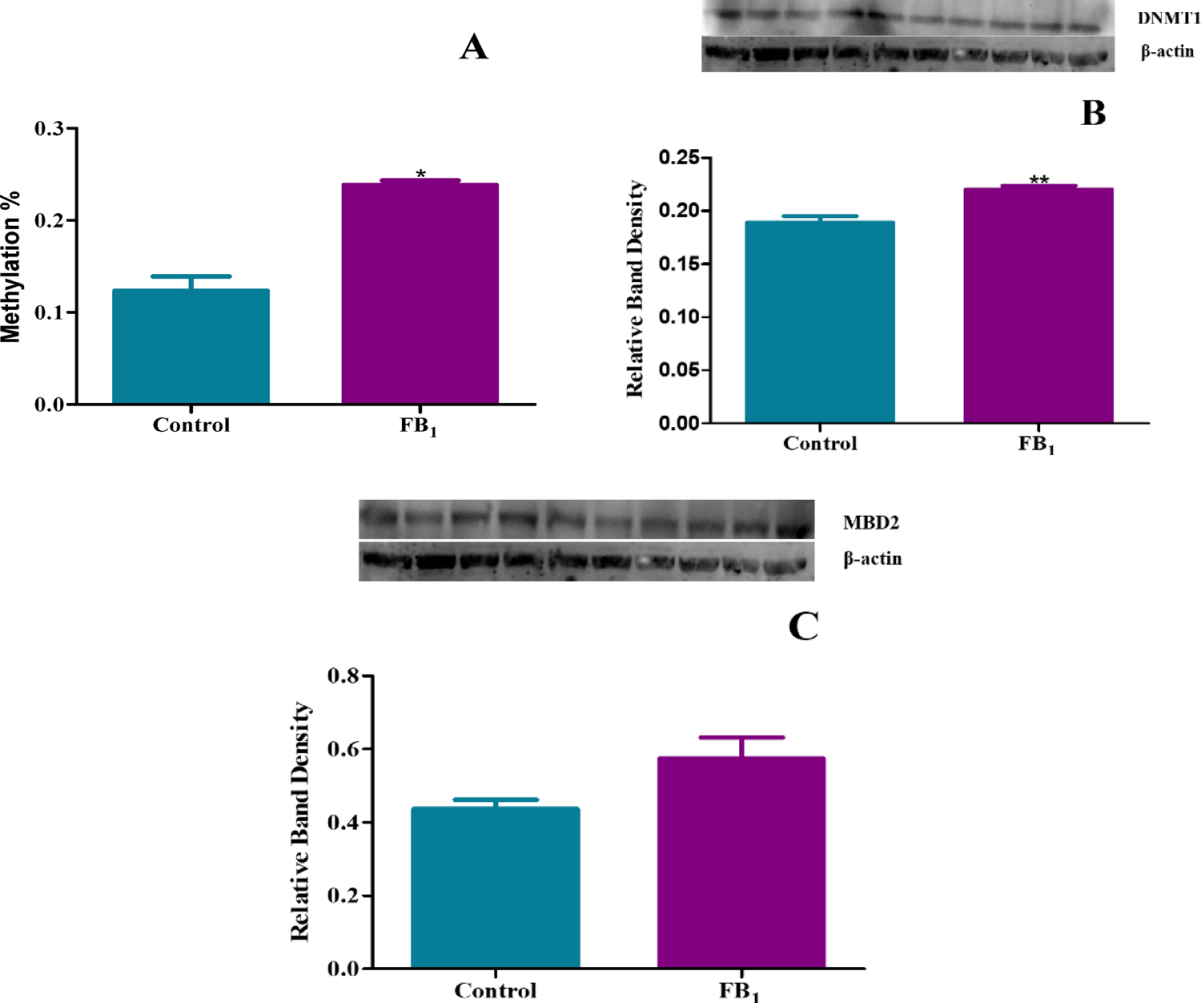
FB_1_ exposure led to increased DNA methylation. The effect of FB_1_ on the protein expression of DNA methylation regulators in C57BL/6 mice hearts. Protein isolated from control and FB1-treated mice hearts as well as *homogenate* was standardised and quantified using ELISA and western blotting. A significant increase in global DNA methylation was observed with FB_1_ treatment (**A**). The protein expression of DNMT1 was significantly upregulated (**B**), while MBD2 was non-significantly increased (**C**). Bands 1 to 5 represent the control, and bands 6 to 10 represent the FB_1_ treatment. The results are shown as mean ± SEM (*n* = 5/group). An unpaired t-test with Welch’s correction was used to determine statistical significance (*p < 0.05; **p < 0.005)

Global DNA methylation levels were found to be significantly increased in mice exposed to FB_1_ with a fold change of 1.93 (*p* = 0.0196) as compared to the controls (Fig. 11A). However, this contrasted with the gene expression analysis of DNA methylation-related genes. The protein expression of DNMT1 was significantly upregulated shown by a fold change of 1.17 (*p* = 0.0036) (Fig. 11B). However, MBD2 a demethylase, depicted a non-significant increase exhibited by a fold change of 1.31 (*p* = 0.0804) (Fig. 11C) in expression.

### Gene expression of DNMTs were decreased with FB_1_ treatment

**Fig. 12:**
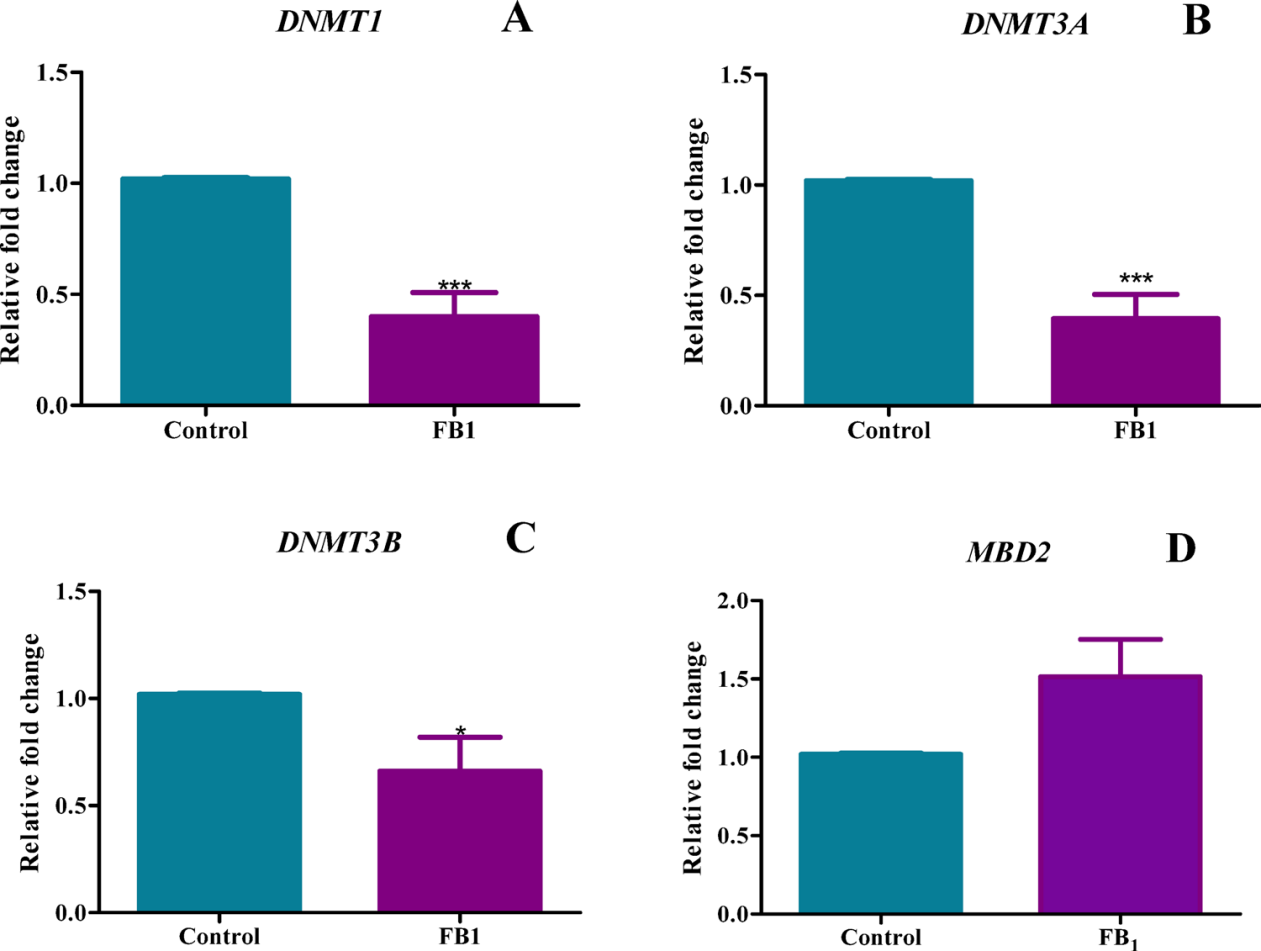
FB_1_ exposure led to dysregulation of DNA methylation-associated genes. The effect of FB_1_ on the gene expression of DNA methylation genes in C57BL/6 mice hearts. FB_1_ exposure led to dysregulation of DNA methylation-associated genes. Gene expression of DNMT1 (**A**), DNMT3A (**B**), and DNMT3B (**C**) were decreased, while MBD2 was non-significantly increased compared to the controls. The results are shown as mean ± SEM (*n* = 5/group). An unpaired t-test with Welch’s correction was used to determine statistical significance (*p < 0.05; ***p < 0.0001)

DNA methylation is an important regulator of gene expression. However, this contrasted with the protein expression analysis of DNA methylation-related factors. Specifically, the gene expression of *DNMT1*, *DNMT3A*, and *DNMT3B* were all significantly decreased [(*p* < 0.0001, percentage decrease = 60.07%), (*p* < 0.0001, percentage decrease = 60.59%), and (*p* = 0.0396, percentage decrease = 33.97%), respectively)] (Fig. 12A–C). For example, *MDB2* (demethylase) showed a non-significant increase represented by a fold increase of 1.51 (*p* = 0.0567) (Fig. 12D), which corresponded with a dysregulation in *DNA methyltransferase* (DNMT) expression. This suggests that FB_1_ exposure has the potential to disrupt gene regulation through altered DNA methylation.

## Discussion

The global prevalence of mycotoxins has been escalating at an alarming rate, posing significant threats beyond economic loss and food insecurity, impacting both human and animal health. Among these fungal secondary metabolites, FB_1_ has emerged as a major contributor to global mycotoxin contamination, with prevalence rates exceeding 80% in countries such as Algeria, Brazil, Nigeria, South Africa, and South Korea (Gao et al. 2023). Despite its increasing toxicity, the cardiotoxic effects of FB_1_ remain inadequately understood, necessitating further research. While toxic effects on organs such as the liver and kidney have been well-documented, studies on FB_1_'s impact on the heart are limited. Previous research has demonstrated that FB_1_ induces oxidative stress, mitochondrial dysfunction, altered sphingolipid metabolism, apoptosis, and epigenetic modifications, including DNA methylation (Merrill Jr et al. 2001, Kim et al. 2018; Gao et al. 2023). However, the cardiotoxic effects and other regulatory pathways, such as inflammation and DNA methylation, require deeper investigation. This pilot study underscores the complex interplay between acute FB_1_ exposure, inflammatory pathways, and DNA methylation, leading to toxic effects in the hearts of mice.

Molecular docking data suggest that FB_1_ has the potential to induce trimerization of the TNF-α protein by facilitating binding within the cleft among its three subunits. Preliminary structural bioinformatics findings indicate that FB_1_ may interact with Tyrosine115 (Tyr115), Proline113 (Pro113), and Serine99 (Ser99) residues on each subunit (Fig. 1). These residues have been highlighted in the literature for their specific functions. The significance of Tyr115 has been suggested as a crucial site for receptor binding in modelling studies, facilitating the activation of the molecule. Similarly, Pro113 may interact with FB_1_ to create hydrogen bonds and interactions that could potentially lead to the inactivation of the molecule (Baeyens et al. 1999). Figure 1 illustrates that FB_1_ possibly binding to TNF-α not only promotes trimerization but also attempts to fit into the groove. This trimerization is essential for the biological activation of TNF receptors, indicating that FB_1_ could theoretically prompt TNF-α activation. However, further structural dynamics studies are necessary to determine the precise contacts and interactions between FB_1_ and the protein, and additional validation of these findings is required.

Following the hypothesized key interaction between FB_1_ and TNF-α in silico, the inducible iNOS, a crucial modulator of the pro-inflammatory response, was docked with FB_1_. The docking analysis suggests that FB1 has a potential affinity for the haeme molecule binding site, facilitating subsequent interaction with cysteine 194 (Cys194) (Fig. 2). Based on this information and the proposed binding position of FB_1_, it is theorised that FB_1_ could enhance the activation of iNOS, leading to alterations in the inflammatory response at an in-silico level (Cinelli et al. 2020). However, further structural dynamics studies using LigPlot + are necessary to elucidate the precise contacts and interactions.

The induction of factors such as TNF-α is believed to subsequently activate NF-κB, which comprises p65 and p50 subunits. Preliminary in silico findings suggest that FB_1_ has a potential affinity for the NF-κB (p60) subunit, specifically interacting with Asn202 and Ser203 residues (Fig. 3). Studies have indicated that mutations or interference at these residues can impair the binding of inhibitor of kappa B alpha (IκBα), leading to the loss of inhibition of the NF-κB molecule (Huxford et al. 2002). In contrast, FB_1_ docking on the p50 subunit of NF-κB revealed interactions with the DNA molecule of NF-κB and the residue Glutamine274 (Gln274) (Fig. 4). Emerging computational studies have highlighted the importance of the p50 homodimer in facilitating specific gene target activation, with dimerization of this subunit assumed to occur due to interactions with Gln274 (Zhu et al. 2023). Given that FB_1_ may interact with the DNA molecule and the p50 subunit at the Gln274 residue, it is postulated that FB_1_ may affect the gene targets of NF-κB. However, these findings are preliminary, and further structural dynamics studies using LigPlot + are necessary to elucidate the precise contacts.

The in-silico findings prompted an examination of the gene expression of inflammatory and DNA methylation factors in vitro. Significant dysregulation in the gene expression of TNF-α, NF-κB, IL-6, NLRP3 inflammasome, IL-18, caspase 1, IL-1β, GSDMD, caspase 3, CT-1, and IL-10 (Figs. 5, 6, 7) was observed in mice treated with FB_1_. As this was a preliminary structural bioinformatics study, it led to predictions that could be tested through experimental methods. These gene expression studies partly aligned with the predictions of the FB_1_-NF-κB in silico docking studies. However, these findings contradicted another study, which showed that FB_1_ upregulated the gene expression of IL-6, IL-1β, TNF-α, (Interferon gamma) IFN-γ, and caspase 3 in porcine alveolar macrophages, with a subsequent increase in serum concentrations of these cytokines (Jin et al. 2022). The observed decline in the expression of both pro-inflammatory and anti-inflammatory genes, as well as associated apoptosis genes, indicated a complex alteration in the inflammation process. Therefore, quantifying the crucial modulator of the inflammatory response, NO, was imperative. The indirect measurement of NO levels using reactive nitrogen species (RNS) in this study depicted an increase in its metabolites, nitrates and nitrites (Fig. 8). Excess production of NO can mediate a heightened pro-inflammatory response, which can be correlated with tissue damage (Sharma et al. 2007).

The observed upregulation of RNS, which was 2.5 times higher than the control, is sufficient to elicit an inflammatory response. Elevated levels of RNS can activate redox-sensitive transcription factors such as NF-κB, leading to increased transcription of pro-inflammatory cytokines and chemokines (Korhonen et al. 2005). These findings align with a study conducted in murine macrophages, which demonstrated that FB_1_ treatment for 24 h resulted in an increase in NO and subsequently peroxynitrite. The study further explored a potential connection between these metabolites and programmed cell death (Lanubile et al. 2022). Given this evidence, it was crucial to further examine the protein expression of pro-inflammatory cytokines.

The analysis of protein expression for key cytokines and inflammation modulators revealed significant upregulation in P-NF-κB, TNF-α, IL-6, IL-1β, IL-10, and TGF-β1 (Figs. 9, 10). This upregulation of pro-inflammatory markers indicates a heightened inflammatory response. Specifically, the increased concentration of TNF-α suggests that FB_1_ exposure triggers the activation of the pro-inflammatory pathway, leading to subsequent activation of P-NF-κB. This, in turn, transcribes the activation of pro-inflammatory mediators IL-6, IL-18, and IL-1β. Concurrently, anti-inflammatory mediators IL-10 and TGF-β1 were also upregulated.

These results are consistent with previous studies conducted in porcine kidney cells, which highlighted the key role of TNF-α in activating inflammatory responses at the protein level, along with upregulated NF-κB and caspase 3 activity, further exemplifying the critical role FB_1_ plays in inflammation-associated apoptosis (Chen et al. 2020). Additionally, a similar study demonstrated the involvement of the cytokine network in FB_1_-mediated toxicity, where cytokines such as TNF-α, IL-1β, IL-6, and IL-10 were concurrently upregulated, mirroring the findings of this study (Bhandari et al. 2001). The concurrent stimulation of IL-10 is crucial for understanding the mediated response, as a study conducted in intestinal tissue showed that IL-10 is a positive regulator of NF-κB pathway transcription, similar to the findings in this study (Papoutsopoulou et al. 2022). Interestingly, a study on mice liver revealed that increased TGF-β1 expression could induce apoptosis due to FB_1_ treatment, correlating with this study’s findings of marked increases in TGF-β1 and caspase 3 expression (Figs. 9, 10) (Lemmer et al. 1999).

The discrepancy observed between the gene and protein expression of inflammatory cytokines could be due to transcriptional or translational modifications. Post-transcriptional modifications play essential roles in biological processes and might explain the differences between gene and protein expression. This is because translational regulation depends on the recruitment of various mRNA species to the ribosome to initiate protein synthesis, leading to a reduced correlation between mRNA levels and the corresponding protein amounts (Halbeisen et al. 2007). Other regulatory mechanisms include epigenetic processes such as DNA methylation, which are critical for modulating gene expression (Poetsch and Plass 2011). Global DNA methylation levels were significantly elevated, with a notable increase in DNMT1 and no significant change in MBD2, indicating DNA hypermethylation (Fig. 11). However, these findings contradicted the expression patterns of DNA methylation genes. Despite the observed DNA hypermethylation, the expression of all DNMT genes significantly decreased, while MBD2 expression showed a non-significant increase (Fig. 12). This suggests a complex interplay, such as mRNA stability, could be a factor. Additional tests, such as Northern blots and microarray assays, can be used to confirm these findings. This study’s results aligned with research conducted on HEK293 cells treated with FB_1_, which showed upregulation of global DNA methylation and DNMT activity (Sugiyama et al. 2021). However, other studies emphasize the role of microRNAs in regulating gene expression. A notable study in cardiomyocytes demonstrated that miR-29b can decrease the gene expression of DNMTs in these cells (Wu et al. 2022). These findings are consistent with this study, further suggesting that complex mechanisms may be involved in gene silencing. Another study on human kidney cells revealed that FB_1_ treatment increased DNA methylation in a set of genes, further implicating FB_1_ in gene modulation (Bayoglu et al. 2024).

Emerging evidence indicates that DNA methylation can significantly influence cytokine activity. A study conducted in bone marrow-derived mast cells demonstrated that DNA methylation can suppress the gene activity of NF-κB, thereby modulating the expression of TNF-α, IL-13, and IL-6. The inhibition of DNA methylation using azacytidine (5-AZA) resulted in increased gene expression of these cytokines, suggesting that further experiments utilizing 5-AZA could be beneficial in investigating the effects of FB_1_ on cytokine gene expression (Li et al. 2024). Similar observations were noted in this study, where FB_1_ treatment led to increased DNA methylation, resulting in gene silencing. This contrasted with the increased protein expression of key cytokines such as TNF-α, NF-κB, IL-6, IL-1β, IL-18, IL-10, and TGF-β1 in mice.

The convergence of these pathways has been shown to contribute to the onset of various cardiac conditions. The pathogenesis of cardiac ailments, such as heart failure (HF), has been associated with elevated levels of TGF-β1, which are activated upon acute ischemia, norepinephrine, or angiotensin II stimulation. Increased TGF-β1 in cardiomyocytes leads to exacerbated fibrosis and hypertrophy in the heart, as evidenced by this study’s depiction of elevated TGF-β1 levels (Fig. 10E) (Osmancik and Louckova 2017). Studies have established that elevated TNF-α in circulation can lead to the development of cardiac hypertrophy. Furthermore, TNF-α has been shown to exert potent effects on the heart by inducing apoptosis and decreasing contractility in vitro (Krown et al. 1996). The complexity of TNF-α signalling, indicative of NF-κB activation, is associated with the pathologies of HF. Given this evidence, cytokines have been shown to be linked with the onset of cardiac distress (Figs. 9A, 10A) (Hilfiker-Kleiner et al. 2006). Other studies indicate that IL-6 is a crucial diagnostic marker for individuals presenting with HF, as IL-6 levels are significantly upregulated (Fig. 10B) (Tsutamoto et al. 1998). In light of this evidence, there is a strong connection between cardiac stress and the inflammatory response. Therefore, it can be concluded that FB_1_ has the potential to affect inflammatory and DNA methylation pathways, potentially leading to cardiac distress.

## Conclusion

This pilot study highlights the potential of FB_1_ to be a potential cardiac distress agent in mice. The results presented in this research shows that FB_1_ dysregulates gene expression of cytokines (*TNF-α, NF-κB, IL-6, NLRP3 Inflammasome, IL-18, caspase, IL-1β, GSDMD, caspase 3, CT-1, IL-10*) however this is contrasted with the associated protein levels which were significantly upregulated (TNF-α, IL-6, IL-1β, IL-10, and TGF-β1). This suggests complex transcriptional modifications such as DNA methylation occurring due to FB_1_. The data obtained from gene and protein analysis furthermore showed a similar dysregulation of (DNMT1, DNMT3A, DNMT3B and MBD2). However significant upregulation of global DNA methylation was present further elucidating other multifaceted regulatory mechanisms responsible for the disparity observed. The convergence of this study heavily indicates the dysregulation in inflammatory and methylation pathways which are characteristic in many cardiac distresses such as heart failure, cardiac fibrosis and hypertrophy.

## Data Availability

The entire data set present, and its relevant supplementary information is obtainable from the corresponding author at a reasonable request.

## Abbreviations

5-AZA: Azacytidine
5mc: 5-Methylcytosine
BCA assay: Bicinchoninic acid assay
CT-1: Cardiotrophin-1
Cys194: Cysteine 194
DNMT1: DNA Methyltransferase 1
DNMT3A: DNA Methyltransferase 3 Alpha
DNMT3B: DNA Methyltransferase 3 Beta
DNMTs: DNA methyltransferases
ELISA: Enzyme-linked immunosorbent assay
FB1: Fumonisin B1
GAFF: General Amber Force Field
GSDMD: Gasdermin D
GSDME: Gasdermin E
Gln274: Glutamine274
HF: Heart failure
IFN-γ: Interferon gamma
IL-10: Interleukin 10
IL-18: Interleukin 18
IL-1β: Inteleukin-1-beta
IL-6: Interleukin 6
IκBα: Inhibitor of kappa B alpha
MBD2: Methyl-CpG-binding domain protein 2
N-GSDMD: N-terminal GSDMD
NEDD: N-(1-Naphthyl) ethylenediamine dihydrochloride
NF-κB: Nuclear factor kappa-light-chain-enhancer of activated B cells
NLRP3 inflammasome: Nucleotide-binding oligomerization domain (NOD) like receptor family pyrin…
NO: Nitric oxide
NOS: Nitric oxide synthase assay
P-NF-κB: Phosphorylated-NF-κB
PBS: Phosphate-buffered saline
Pro113: Proline113
qPCR: Quantitative polymerase chain reaction
RBD: Relative band density
RMSD: Root mean square deviations
RNS: Reactive nitrogen species
SEM: Standard error of the mean
Ser99: Serine99
TMB: 3,3′,5,5′-Tetramethylbenzidine
TNF-α: Tumour necrosis factor alpha
Tyr115: Tyrosine115
